# Dietary fiber intake and hippocampal gray matter volume: an exploratory cross-sectional study in healthy adults

**DOI:** 10.3389/fnut.2025.1608995

**Published:** 2025-06-16

**Authors:** Raghav Pallapothu, Roger D. Newman-Norlund, Makayla Gibson, Pranesh Rajesh Kannan, Sriya Pallapothu, Chris Rorden, Leonardo Bonilha, Julius Fridriksson

**Affiliations:** ^1^Department of Psychology, College of Arts and Sciences, University of South Carolina, Columbia, SC, United States; ^2^Department of Neurology, School of Medicine, University of South Carolina, Columbia, SC, United States; ^3^Department of Communication Sciences, Arnold School of Public Health, University of South Carolina, Columbia, SC, United States

**Keywords:** brain, gray matter volume, fiber, Alzheimer’s disease brain, dementia

## Abstract

**Introduction:**

Alzheimer’s disease and related dementia (ADRD) are associated with significance societal costs and economic burdens. Prior research has found that ADRD is associated with decreases in cortical gray matter volume (GMV) in the hippocampus.

**Methods:**

This exploratory cross-sectional study investigates the relationship between total dietary fiber concentration and regional GMV in a cohort of 190 healthy adults aged 20 to 79 years who completed the 2015 National Health Interview Survey Supplement and T1-weighted magnetic resonance imaging (MRI) as part of the University of South Carolina’s Aging Brain Cohort (ABC) study. Hippocampal gray matter volume was quantified using the CAT12 voxel-based morphometry toolbox, using Spearman’s rank order correlation coefficient.

**Results:**

Our exploratory analysis revealed a significant positive correlation between dietary fiber consumption and GMV in the right hippocampus and right parahippocampal gyrus, when controlling for age, race, sex, and median household income within participant zip code. Dietary fiber was also correlated with the MoCA attention/concentration and language subscores.

**Discussion:**

These data are compatible with the hypothesis that dietary fiber may exert neuroprotective effects, and this may have implications for dietary recommendations aimed at reducing the risk of Alzheimer’s disease and related dementias.

## Introduction

Dementia is one of the most common neurological disorders, with over 55 million people worldwide living with this diagnosis in 2020 and over 10 million new cases occurring annually (1). Alzheimer’s disease, the most prevalent form of dementia, is typically characterized by an abnormally large loss of gray matter across the brain, including areas of the hippocampal and temporal regions (2). Thus, gray matter, responsible for memory functions, emotional regulation, movement, and muscle control, is seemingly linked as a potential biomarker of neurodegenerative disorders like Alzheimer’s.

The hippocampus is particularly vulnerable to gray matter loss in neurodegenerative disorders including Alzheimer’s, which is characterized by symptoms such as memory loss and cognitive decline (3). It has been well established that hippocampal atrophy is strongly correlated with Alzheimer’s disease and dementia progression, with reductions in hippocampal gray matter volume preceding significant cognitive impairment (4).

Recent findings suggest that diet, particularly dietary fiber intake, may play a significant role in protecting hippocampal integrity and function. The gut microbiota, which is heavily influenced by fiber intake, plays a crucial role in modulating neuroinflammation and cognitive function through the gut-brain axis. A diet deficient in fiber has been shown to alter gut microbiota composition, leading to systemic inflammation and reduced production of short-chain fatty acids (SCFAs) like butyrate, which have neuroprotective properties (5).

In a recent study using a mouse model of long-term fiber deficiency (FD), sustained low fiber intake was associated with significant impairments in cognition, including deficits in object location memory and temporal order memory — functions primarily mediated by the hippocampus. Additionally, the study found that hippocampal synaptic ultrastructure was negatively affected, leading to decreased synaptic plasticity and weakened neuronal connectivity, both of which are hallmarks of cognitive decline in aging and neurodegenerative diseases. Furthermore, the FD mice exhibited increased neuroinflammation within the hippocampus, suggesting that fiber deficiency promotes a pro-inflammatory state that exacerbates gray matter loss (6). Although similar studies in humans have not been conducted, research indicates that diets rich in fiber have been associated with larger hippocampal gray matter volumes and better cognitive performance in aging populations. The mechanisms underlying these benefits are thought to involve a combination of reduced systemic inflammation and improved blood–brain barrier integrity (7).

Given what is known about the relationship between diet and brain health, this exploratory cross-sectional study sought to specifically examine the hypothesis that brain health would be associated with a healthy diet in healthy adults. Specifically, we predicted that hippocampal gray matter volume would be correlated with total dietary fiber in a convenience sample of 190 participants from the Aging Brain Cohort (ABC@USC) study.

## Methods

This study was an exploratory cross-sectional design that used a self-reported survey: the National Health Interview Survey Supplement 2015 Survey (8), a 31-question survey administered every 5 years. Total and regional gray matter volumes were derived from T1-weighted magnetic resonance imaging (MRI) scans. Ethical approval for this study was obtained from the University of South Carolina institutional review board. All methods were performed in accordance with the relevant guidelines and regulations, ensuring adherence to ethical standards for research involving human subjects (9), and informed consent was obtained from all participants prior to participation in the study.

### Participants

Data from the NHIS 2015 Dietary Questionnaire was drawn from the University of South Carolina’s Aging Brain Cohort Study Repository, a multimodal database (10). As part of this study, T1-weighted MRI images were collected. From the duration of September 2019–December 2023, participants underwent an MRI scan using the University of South Carolina T1-weighted structural MRI scanner. The University of South Carolina IRB approved this procedure. The 190 participants were between 20 and 79 years old (Mean age = 48.284, 49 males, 141 females). To remain in suitable conditions for testing, participants had to stay in a supine position for nearly an hour, with their maximum girth being lower than 60 inches and maximum weight under 400 pounds. Participants did not have existing medical conditions including, but not limited to: severe illnesses like cancer, untreated and unmanaged psychological conditions like schizophrenia, and no current or past fatiguing illnesses. In this study, the number of participants was determined by the amount of data available for analysis as all available data was utilized. This dataset specified the sex, age, median household income within zip code, race, and handedness of each individual as fixed variables.

### Scanning protocol

All participants underwent the same magnetic resonance imaging (MRI) scanning protocol at the University of South Carolina, McCausland Center for Brain Imaging on a Siemens Trio 3T scanner with a 20-channel head coil. T1-weighted images were used for volumetric analyses and were acquired using the following parameters: T1-weighted imaging (MP-RAGE) sequence with 1 mm isotropic voxels, 256 × 256 matrix size, 9° flip angle, and 92-slice sequence with repetition time = 2,250 ms, inversion time = 925 ms, and echo time = 4.11 ms. The entire scanning protocol is described in the ABC repository paper (10).

### Voxel-based morphometry (VBM) analysis

To investigate the structural differences in gray matter, we utilized the Computational Anatomy Toolbox (CAT12), running in SPM12 and Matlab 2024b. CAT12 supports voxel-based morphometry (VBM) using a fully automated workflow (11). Standard processing steps included application of bias correction to minimize MRI artifacts, followed by brain segmentation into gray matter (GM), white matter (WM), and cerebrospinal fluid (CSF) using standard tissue classification algorithms. The preprocessing protocol included spatial registration to a reference brain, tissue segmentation, and correction for intensity non-uniformities. To specifically isolate the hippocampus and parahippocampal regions, we employed the AAL3 brain parcellation scheme during preprocessing (12). These steps facilitated the accurate estimation of gray matter volume (GMV) across different cortical regions. The regional GM volumes were then normalized to the total brain GMV to assess the proportional distribution within the cortex. Statistical analyses were performed using JASP open source software (13) to detect structural variances across different groups using the processed data. We conducted our primary VBM analyses using Spearman’s correlations between region-level gray matter density and dietary fiber intake. Since all four statistical correlations between GMV and dietary fiber intake were planned apriori, we did not apply corrections for multiple comparisons, focusing instead on the robustness of the associations identified.

### Fiber concentration calculation

The concentration of fiber was calculated as the proportion of grams of fiber out of the total grams of food per serving using this source (14). If certain items were not found on the USDA, the University of Rochester Medical Center Adult and Children’s Health Encyclopedia was utilized (15). However, when cereals were evaluated, the SmartLabel (for Kellogg’s products) and box labels were utilized to estimate the most accurate fiber concentration of each item.

Prior studies have shown that dietary fiber concentration can vary significantly within a given category, such as whole grains versus refined grains, or raw versus cooked vegetables. To account for this, we used a weighted average approach that prioritizes foods with more frequent consumption within a category, thereby improving the precision of our estimates.

In the question “How often did you eat CHOCOLATE, or any other types of CANDY? Do NOT include SUGAR-FREE CANDY” on the NHIS 2015, the 20 most popular chocolates and candies by market share, sales volume, and keyword traffic, were chosen and averaged (Afzal, 2023). For the question “How often did you eat WHOLE GRAIN BREAD including toast, rolls and in sandwiches? Whole grain bread includes whole wheat, rye, oatmeal and pumpernickel,” the USDA fiber concentration values of whole wheat, rye, oatmeal, and pumpernickel were averaged to input single fiber-concentration for whole grain bread. A similar procedure was followed for the “How often did you eat COOKIES, CAKE, PIE, or BROWNIES?” question. For the NHIS 2015 question “How often did you eat POPCORN?,” three representative classic-type popcorn brands (Act ii, Pop Secret, Orville Redenbacher) were averaged to output average fiber concentration for the popcorn variable. Finally, for the question “How often did you drink COFFEE or tea that had sugar or honey added to it? Include coffee and tea you sweetened yourself and presweetened tea and coffee drinks such as Arizona Iced Tea and Frappuccino,” a Starbucks Coffee Frappuccino with whole milk, Arizona Lemon Iced Tea, a Starbucks Medium Pike Place Roast, and a Starbucks Earl Gray Tea were averaged to find the fiber concentration.

In the study, participants had the option to input the frequency with which they consumed a food. The possible frequency responses were given this number scale from 1–9: Never (1), 1-time last month (2), 2–3 times last month (3), 1 time per week (4), 2 times per week (5), 3–4 times per week (6), 5–6 times per week (7), 1 time per day (8), and 2 or more times a day (9). Table 1 summarizes the demographic details of the 190-subject dataset utilized in this study.

**Table 1:** Demographic variables of interest.

Category	Measure	Valid	Miss	M ± (SD)	Min	Max	Range
Demographics	Race	190	0	W (153), AA (27), A (9), NA (1)			
	Sex	190	0	N (M) = 49, N (F) = 141			
	Age	190	0	48.284 ± 19.804	20	79	59
	Income	158	32	45468.42 ± 35333.334	1,250	175,000	173,750
	Hand	155	35	R (139), L (12), A (4)			

W, White; AA, African American; A, Asian; NA, Native American. R, Right-handed; L, Left-handed; A, Ambidextrous. Miss, Missing/unavailable data; M, Mean; SD, Standard Deviation; Min, Minimum; Max, Maximum; N (M), Number of male participants; N (F), Number of female participants; Hand, handedness.

### Statistical methods

To properly deduce correlations among fiber concentration, an index that accounts for food consumed and its frequency was created and is defined as such: each food group was calculated with a fiber concentration. The frequency of consumption inputted by the users for a question was then multiplied by the established fiber concentration. This operation was completed for all 31 questions. Then, the 31 fiber indexes were independently summed to create a distinct fiber-concentration index (total fiber) for the entire user-consumed diet.

Following the data sorting, we examined correlations between total fiber concentration and regional gray matter volume using JASP (13). Given that the dietary index data are ordinal, based on frequency inputs, and may not follow a normal distribution, Spearman’s method provides a more accurate correlation measure compared to Pearson’s correlation. Furthermore, Spearman’s correlation is less affected by outliers, which is advantageous when working with diet-related data that can have extreme or skewed values.

## Results

Complete data on demographic factors including age, sex, and race were available for all 190 analyzed subjects in the dataset. However, median household income per zip code was only available for 158 of these participants, and handedness was only available for 155 participants. The results in Table 2 reveal several noteworthy correlations between higher fiber consumption and increased gray matter, particularly in areas involved in executive function and emotional processing. Only the 158 participants with full demographic data were used in the final analyses that included brain and MoCA data.

**Table 2:** Spearman’s rho for demographics and variables of interest.

Variable	Value	Age	Sex	Race	Inc	Hand
Total dietary fiber concentration	*n*	190	190	190	158	155
	Spearman’s rho	**0.219**	0.102	**−0.158**	0.154	0.015
	*p*-value	**0.002**	0.162	**0.029**	0.054	0.425

Correlations between total dietary fiber concentration in diet and demographic factors of age, sex, race, and income. Race was quantified as 1 (White), 2 (African American), and 3 (Other Race). Handedness was quantified as 0 (Right-handed), 1 (Left-Handed), and 2 (Ambidextrous). Methods detail how total dietary fiber was calculated. Significant correlations are bolded (p<0.05). Inc, Median household income within zip code; *n*, sample size; Hand, handedness.

### Correlations between hippocampal integrity and fiber content

**Figure 1:**
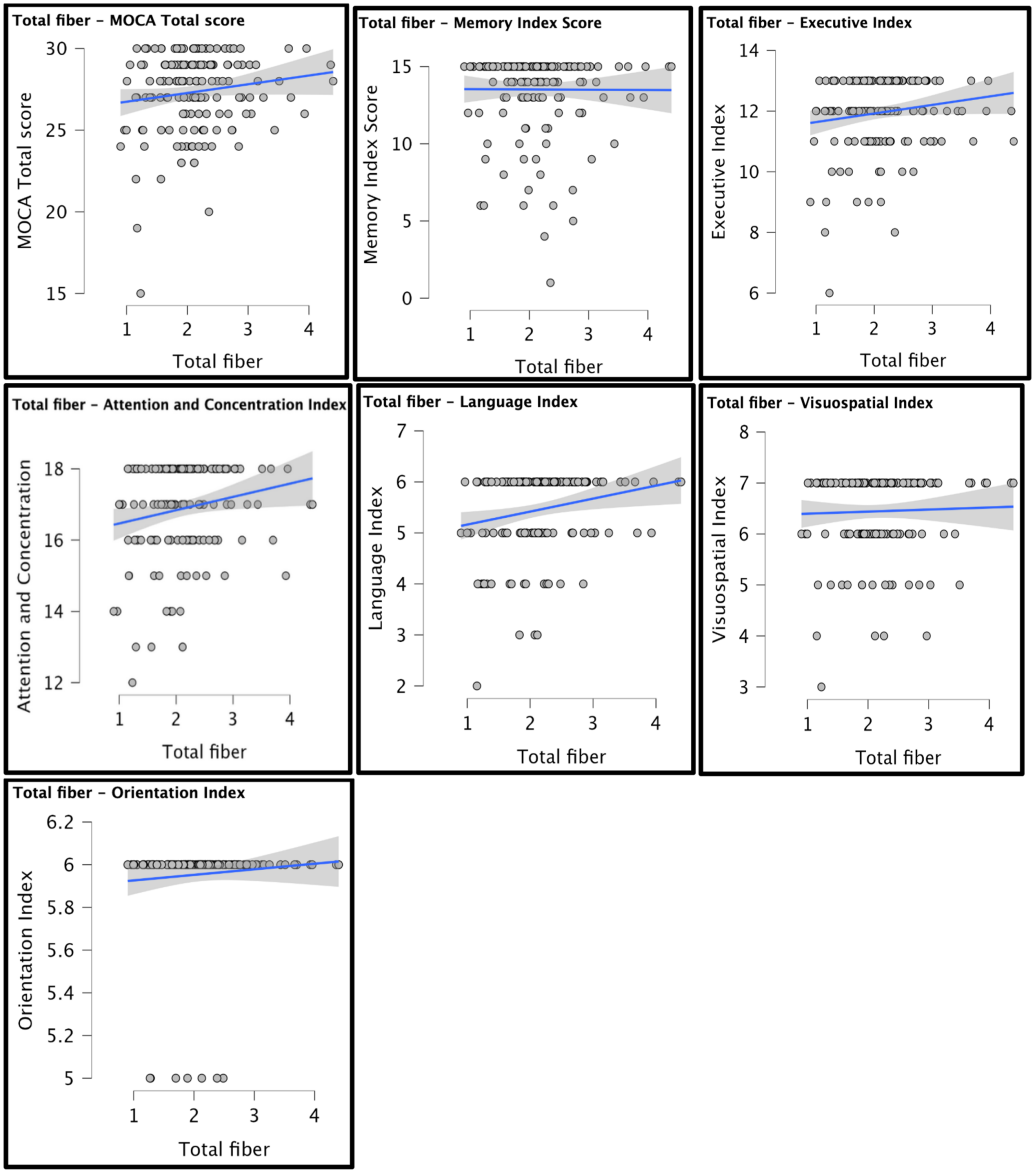
Scatterplots of total fiber and MoCA scores are depicted. Each graph shows a line of best fit to understand the overall estimated trend in the data. Demographic variables were not controlled for in the scatter plot analysis.

**Figure 2:**
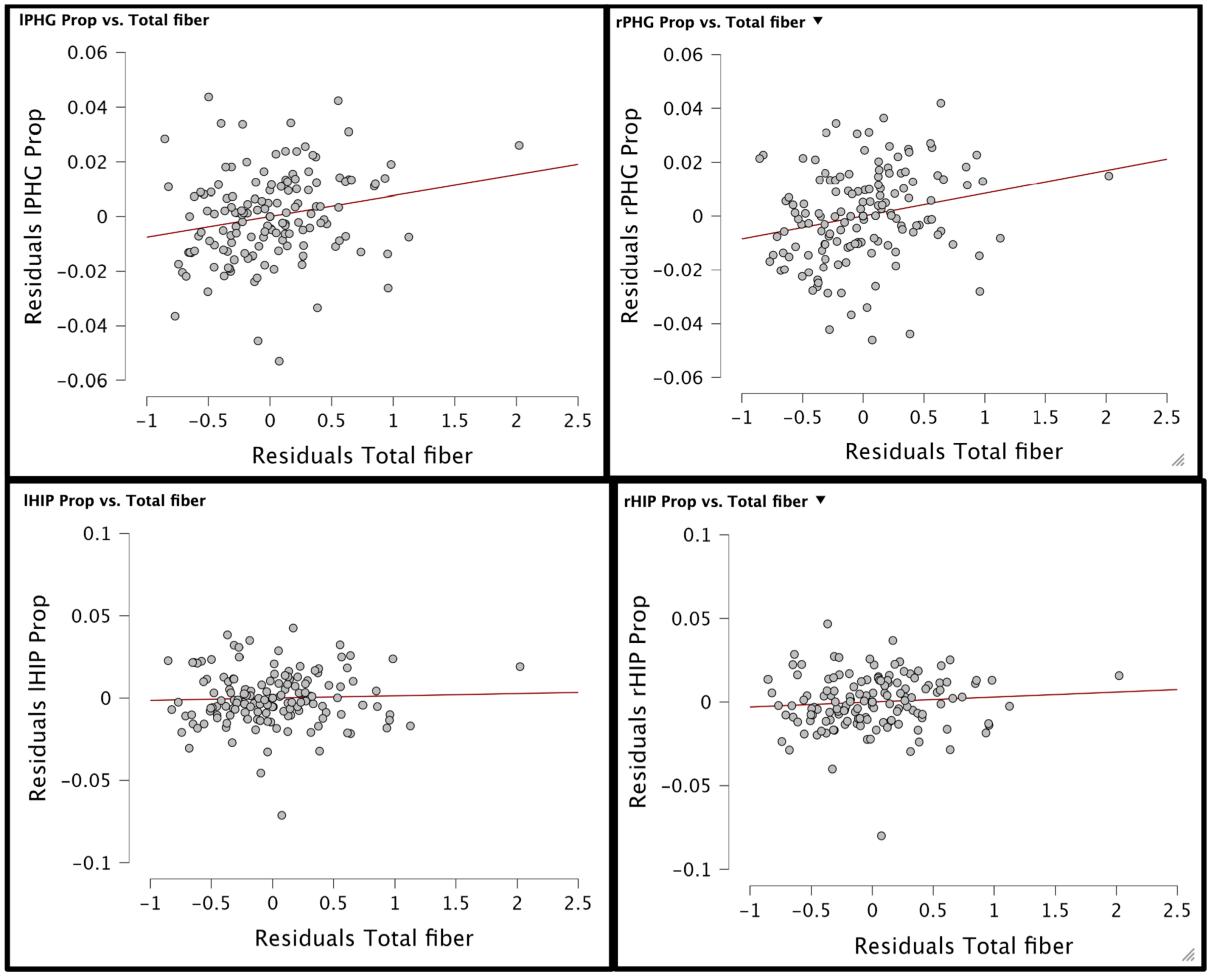
Residual plots of total fiber diet concentration and gray matter volume of 4 hippocampal brain areas have been generated. Top shows residual plot of lPHG/rPHG gray matter volume proportion and total fiber concentration while bottom shows residual plot of lHIP/rHIP gray matter volume proportion and total fiber concentration in diet.

**Figure 3:**
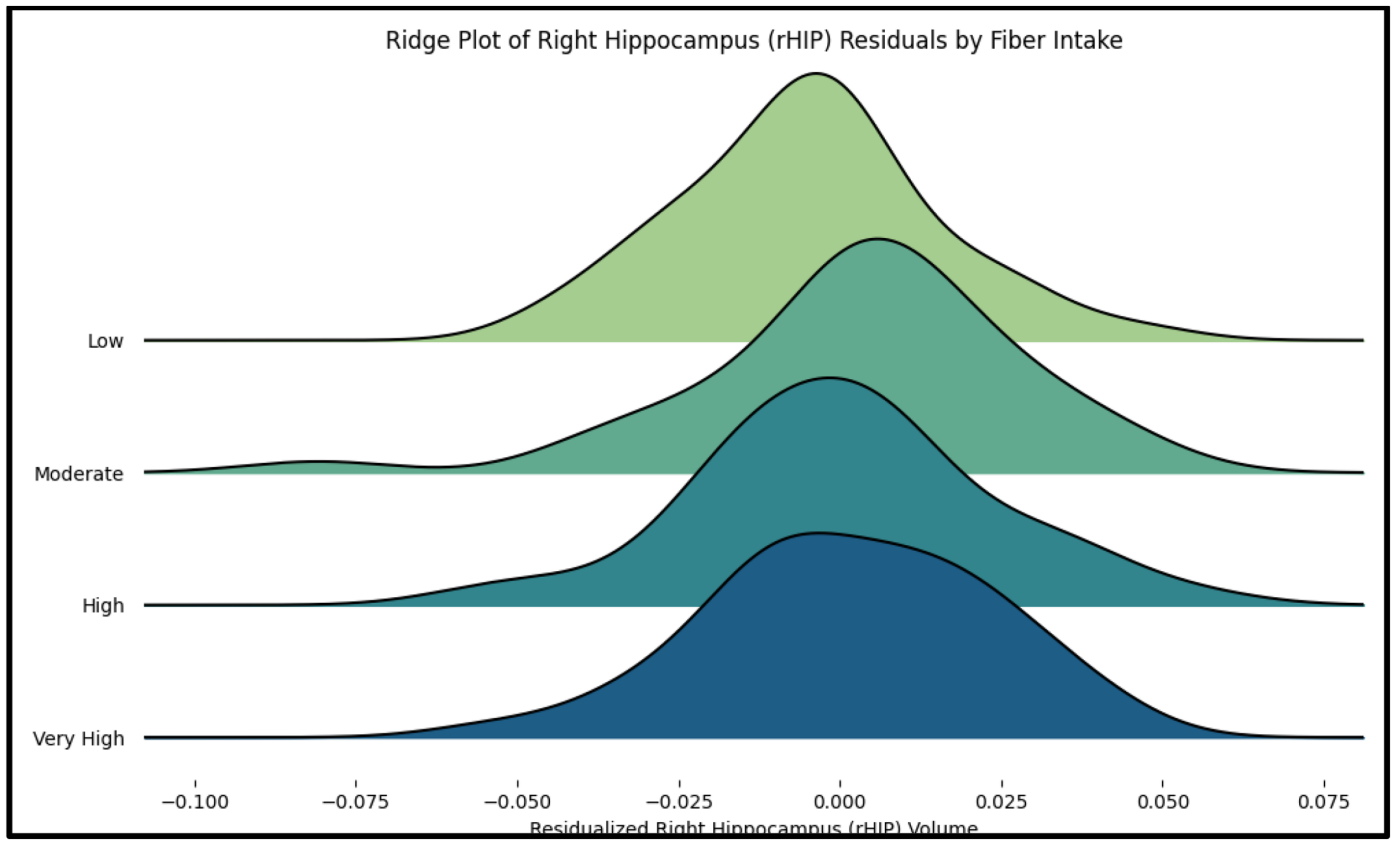
The ridge plot displays the distribution of residualized right hippocampus (rHIP) volume across quartiles of fiber intake (Low, Moderate, High, Very High). Each curve represents the distribution of rHIP volume residuals, adjusted for age, sex, race, and socioeconomic status (SES).

A series of planned one-tailed Spearman’s correlations examined the relationship between dietary fiber and cognitive scores (MoCA total scores, Language/Attention and Concentration Index) and controlled for age, sex, race, and median household income within zip code. Table 3 depicts these results and Figure 1 depicts scatterplots of the relationship between total fiber consumption and each MoCA subscore. A second series of one-tailed Spearman’s correlations were used to assess the relationship between dietary fiber intake and gray matter volume in bilateral hippocampal and parahippocampal regions of interest while controlling for demographics (age, sex, race, median household income within zip code, and handedness). A residual plot of these results are pictured in Figure 2. Figure 3 depicts an analysis of different fiber-concentrated diets and a residual plot of rHIP (right hippocampus) GMV for a diet with low fiber (0.90 to 1.74 index), moderate fiber (1.74 to 2.09 index), high fiber (2.09 to 2.41 index) and very high fiber (2.41 to 4.40 index). The quartiles were calculated for each to include approximately 25 percent of the participant fiber intakes. Our results revealed higher GMV in the right hippocampus (rHIP) [r (155) = 0.140, *p* = 0.044] and right parahippocampal gyrus (rPHG) [r (155) = 0.147, *p* = 0.037] was associated with higher fiber consumption.

**Table 3:** Dietary factors and their association with MoCA scores.

Variable	Value	MoCA Tot	Mem	Exec	Att/Conc	Lang	Visuo Spa	Orien
Total fiber	*n*	158	158	158	158	158	158	158
	Spearman’s r	0.114	0.009	0.097	0.160	0.138	0.002	0.086
	p-value	0.08	0.458	0.115	**0.024**	**0.043**	0.489	0.145

Correlations between dietary fiber and total fiber in diet. Methods detail ways in which total fiber of diet was calculated and analyzed using a Spearman’s correlation test. *n*, sample size; MoCA Tot, MoCA Total Score; Mem, Memory Score; Exec, Executive Index; Att/Conc, Attention and Concentration Index; Lang, Language Index; Visuo Spa, Visuospatial Index; Orien, Orientation Index. Bolded *p*-values indicate statistical significance (*p* < 0.05) for the association between total dietary fiber and MoCA subdomains, specifically the Attention and Concentration Index and the Language Index.

## Discussion

This exploratory cross-sectional study explored the relationship between dietary fiber intake and GMV in the hippocampus in a healthy population. Within this population, our data showed that higher dietary fiber consumption is positively correlated with GMV in hippocampal areas, supporting the hypothesis that a diet rich in fiber can improve GMV.

Our study extends the findings of the mouse model of dietary fiber deficiency (FD) by providing evidence of a positive correlation between high fiber intake and gray matter volume in key brain regions such as the right hippocampus (rHIP) and right parahippocampal gyrus (rPHG). In prior rodent research, FD mice exhibited cognitive impairments and alterations in synaptic ultrastructure, with a notable dysbiosis in gut microbiota and neuroinflammation. Our results extend these findings by showing that increased dietary fiber intake in humans correlates with higher gray matter volumes in regions critical for memory and cognition. One tantalizing possibility is that high fiber diets exert a protective effect against the cognitive deficits observed in the FD mice, as suggested by previous literature in human beings (16). The rodent study highlighted the detrimental effects of a fiber-deficient diet on hippocampal synaptic structure and cognitive function. Our findings are consistent with this and furthermore suggest a bare minimum level of fiber may be sufficient to promote hippocampal health (Figure 3). Specifically, individuals with at least 1.74 dietary fiber index score (calculations described in Methods above) had higher GMV than individuals with lower values. Our findings in Figure 3 also suggest the presence of a ‘Goldilocks effect,’ where participants consuming diets with moderately dense fiber content exhibited slightly greater right hippocampal GMV compared to those with either low or extremely high fiber intake. This pattern indicates that while fiber is generally beneficial, excessive intake may diminish its positive effects, aligning with the idea that an optimal range of fiber consumption may best support hippocampal health. The exact mechanisms by which fiber intake impacts GMV are still unknown, although prior research has proposed that gut-brain axis interactions may play a role (6). While our study did not assess gut microbiota or inflammatory markers directly, this remains a promising mechanistic hypothesis that warrants further investigation in future work.

Our significant correlations emerged predominantly in the right hippocampus and right parahippocampal gyrus. One possibility is that while the left hemisphere is classically dominant for language, the right medial temporal lobe may be more involved in visuospatial memory and contextual processing: functions that are mainly associated with the hippocampus (17). This lateralization may help explain the right-sided findings, though further research is needed to understand the neurobiological basis of hemispheric asymmetry in the context of nutrition and GMV.

Interestingly, fiber intake was primarily driven by cereal intake in the current study, suggesting that breakfast choices played a dominant role in participants’ overall fiber intake. This finding highlights the potential influence of morning dietary habits in shaping long-term fiber consumption patterns. This suggests that interventions designed to increase fiber intake might best target cereal option choices.

### Dietary alterations and hippocampal gray matter volume

**Figure 4:**
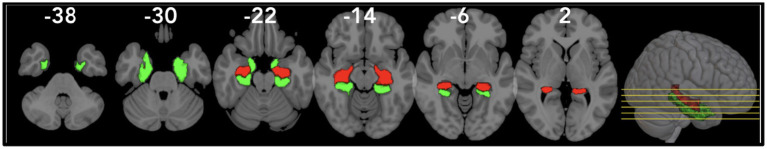
Visualization of the right/left Parahippocampal Gyrus (rPHG/lPHG) and right/left Hippocampus (rHIP/lHIP) using the AAL3 atlas in MNI space. The rPHG/lPHG (red) and rHIP/lHIP (green) are shown in views to highlight their anatomical positioning.

Based on prior rodent research, we predicted that hippocampal GMV would be a sensitive biomarker for neurodegenerative disorders and an indicator of cognitive function in our sample. Specifically, higher dietary fiber intake has been linked to improved cognitive performance, including faster information processing and better working memory (18). Our study builds on this by showing that a higher fiber-concentrated diet is associated with increased GMV in specific brain regions, particularly areas linked to cognition (19). Figure 4 illustrates this relationship, highlighting the right hippocampus and right parahippocampal gyrus as regions where GMV was significantly associated with fiber intake. Notably, previous research has often overlooked structural brain differences like GMV or localized areas influenced by dietary changes, making our findings significant as they specifically highlight the impact of fiber intake on GMV in localized brain regions, specifically the hippocampus.

### Supplemental data

**Figure 5:**
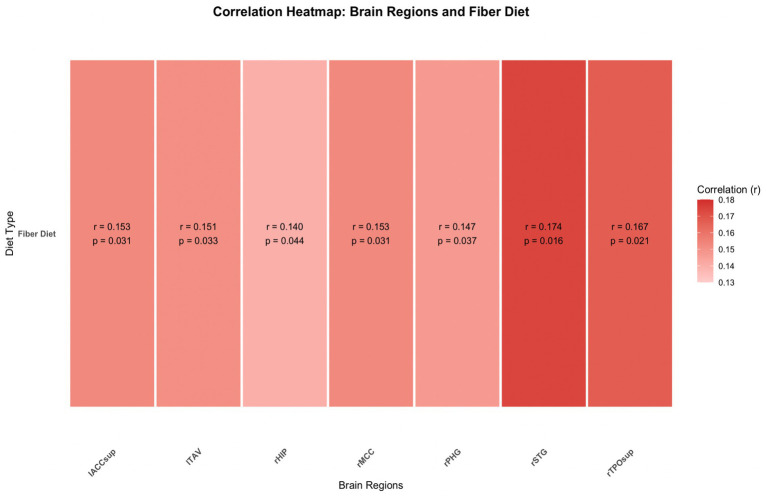
The correlation gray matter volume of eight brain regions with total fiber consumption in diet is depicted above. The correlated heatmap utilizes results depicted in the supplementary data.

Expanded results of the complete one-tailed Spearman’s correlation test are depicted in the supplemental data. Due to existing literature suggesting the cognitive benefits of fiber concentration, the alternative hypothesis was that the factors of fiber concentration in diet and GMV were correlated positively. The following areas yielded results including: right Middle Cingulate Cortex, rMCC, [r (155) = 0.153, *p* = 0.031]; right Hippocampus, rHIP, [r (155) = 0.140, *p* = 0.044]; right Parahippocampal Gyrus, rPHG, [r (155) = 0.147, *p* = 0.037]; right Superior Temporal Gyrus, rSTG, [r (155) = 0.174, *p* = 0.016]; right Temporo-Parietal Occipital Junction (superior), rTPOsup, [r (155) = 0.167, *p* = 0.021]; left Temporal Anterior Ventral area, lTAV, [r (155) = 0.151, *p* = 0.033]; Intra-Anterior Cingulate Cortex, superior part, IACCsup, [r (155) = 0.153, *p* = 0.031]. Figure 5 depicts these results in a heatmap format.

However, only the right hippocampus and right parahippocampal gyrus were part of the *a priori* region of interest (ROI) analysis and thus could be interpreted without correction for multiple comparisons. The remaining regions were explored *post hoc* and are presented in the Supplementary data for context, but not as formal results. Because corrections for multiple comparisons were not applied beyond the predefined ROIs, these exploratory findings may increase the likelihood of type I errors. As such, they should be interpreted with caution and seen as preliminary observations warranting further investigation.

### Limitations and future directions

One limitation of the current study is its relatively low sample size compared to prior studies relating hippocampal volume to cognition. For example, past studies conducted on fiber-concentrated diets and their impact on cognition typically utilized a N≥1000: a significantly larger number of participants than available at the University of South Carolina Aging Brain Cohort. This study should be replicated to ensure our findings were not spurious. A clear method for future research would be to obtain MRI data from larger de-identified clinical databases like the ADNI Alzheimer’s database (20) where participants also have dietary data through questionnaires like the NHIS 2015 Dietary Questionnaire. Additionally, the sample was disproportionately female (141 females vs. 49 males), which may introduce bias and limit the generalizability of our findings. Given that gray matter volumes and dietary patterns can differ by sex, this imbalance could have influenced both the observed correlations and their strength. Future studies should aim for more balanced gender representation to ensure more robust and generalizable conclusions.

Additionally, although race, age, median household income within zip code, and demographic variables were accounted for during the analysis, other variables such as mental well-being, sleep duration, and physical activity levels were not collected nor controlled for during the analysis. Furthermore, we used household income as a measure of socioeconomic status, which is a relatively crude metric. Referring to Figure 1, the residual plot analysis demonstrates that there is some confounding discrepancy in both analyses, particularly the analyses of fiber concentration and GMV proportion in the rPHG. Another limitation of this study is the reliance on self-reported dietary intake from the NHIS 2015 questionnaire, which may introduce recall bias and measurement error. The absence of complementary dietary data, such as caloric intake, macronutrient composition, or food diversity, further restricts our ability to control for confounding variables. Additionally, the lack of validation through objective measures like food diaries or dietary biomarkers limits the precision of our fiber intake estimates. Future studies should incorporate these tools to improve accuracy and better characterize the relationship between diet and brain structure.

Other variables such as sleep duration, physical activity, and chronic diseases like diabetes were not collected nor controlled for during the analysis. Previous literature has stated that higher physical activity and exercises have been linked to greater GMV in the hippocampus, as the volume of the hippocampus remains responsive to moderately intense exercises for 6–12 months (21). In addition, one source states that the optimal sleep duration associated with peak GMV is 6.7–7 hours, indicating that subjects who sleep approximately this long could naturally have a larger GMV than their counterparts regardless of dietary changes (22). Thus, we were not able to control for a large amount of confounding variables beyond age, race, socioeconomic status, and handedness in our study, possibly resulting in skewed data.

## Conclusion

Our data provides novel evidence that, in humans, fiber-concentrated diets calculated from the NHIS 2015 Dietary Questionnaire may be linked to increased localized GMV in areas related to memory, i.e., the hippocampus. Given the increasing societal costs of Alzheimer’s disease and related dementias, targeting dietary fiber intake could be a promising intervention for slowing the progression of neurodegenerative disorders like Alzheimer’s disease in elderly populations. It is suggested that future studies focus on how other macronutrients like carbohydrates, proteins, and fats contribute to GMV. Future studies should also aim to develop increasingly sophisticated nutrition trackers to more precisely record the total fiber in the diet and investigate the use of linear regression machine learning models to predict dietary effects on brain health and the development of neurodegenerative diseases.

## Data Availability

The raw data supporting the conclusions of this article will be made available by the authors, without undue reservation.
