# Supplementary material for: Dietary fiber intake and hippocampal gray matter volume: an exploratory cross-sectional study in healthy adults
Source: Front Nutr. 2025 Jun 16;12:1608995. doi: 10.3389/fnut.2025.1608995 (PMC12206789; doi:10.3389/fnut.2025.1608995)
Supplement: Supplementary file 1 [file Table_1.DOCX]

**Supplementary** Associations between total fiber consumption and localized GMV

| **Variable** | **Value** | **rMCC Prop** | **rHIP Prop** | **rPHG Prop** | **rSTG Prop** | **rTPOsup Prop** | **ltAV Prop** | **lACCsup Prop** |
| --- | --- | --- | --- | --- | --- | --- | --- | --- |
| Total Fiber | n | 155 | 155 | 155 | 155 | 155 | 155 | 158 |
|  | Spearman’s r | **0.153** | **0.140** | **0.147** | **0.174** | **0.167** | **0.151** | **0.153** |
|  | p-value | **0.031** | **0.044** | **0.037** | **0.016** | **0.021** | **0.033** | **0.031** |

Note: Correlations between total fiber and diet and various areas of the brain. The localized gray matter volume of eight distinct areas of the brain were seen to have significant associations with total fiber in diet. Methods detail ways in which total fiber of diet was calculated and analyzed using a Spearman’s correlation test. Abbreviations: n, sample size; rMCC, right Middle Cingulate Cortex; right Hippocampus, rHIP; right Parahippocampal Gyrus, rPHG; right Superior Temporal Gyrus, rSTG; right Superior Temporal Pole, rTPOsup; left Anterior Ventral Insula, ltAV; left Superior Anterior Cingulate Cortex. lACCsup.
